# YouTube as a source of information on endoscopic retrograde cholangiopancreatography

**DOI:** 10.1097/MD.0000000000030724

**Published:** 2022-09-23

**Authors:** Hoonsub So, Do Won Kim, Jun Seong Hwang, Sung Woo Ko

**Affiliations:** a Department of Internal Medicine, Ulsan University Hospital, University of Ulsan College of Medicine, Ulsan, Republic of Korea; b Center of Hepatobiliary & Pancreatic diseases, Department of Internal Medicine, Hanyang University College of Medicine, Changwon Hanmaeum Hospital; c Department of Internal Medicine, Eunpyeong St. Mary’s Hospital, Catholic University of Korea, Seoul, Republic of Korea.

**Keywords:** endoscopic retrograde cholangiopancreatography, YouTube

## Abstract

Endoscopic retrograde cholangiopancreatography (ERCP) carries a higher risk of adverse events than standard endoscopy. Internet media platforms such as YouTube has emerged as a medical information source. Therefore, study aimed to identify whether YouTube videos provide appropriate information on ERCP to the general population. The YouTube search was performed using the terms “endoscopic retrograde cholangiopancreatography” and “ERCP”. The top 50 results of both searches, sorted by relevance and view count, were collected. After filtering according to a set of inclusion and exclusion criteria, a total of 26 videos were eligible for the final analysis. For quality assessment, we created a scoring system called ERCP Data Quality score (E-DQS), based on a colonoscopy education video available on the American Society of Gastrointestinal Endoscopy website. Healthcare professionals uploaded 14 (53.8%) videos, and 10 (38.6%) uploaded by medical websites. Only one video was uploaded by a layperson and one by a TV channel. The overall median E-DQS score for enrolled videos was 6.5 out of 20. The majority of videos did not describe the unique features of ERCP. Only 50% of videos informed viewers that patients would be irradiated and only six videos described at least one adverse event related to ERCP. ERCP videos on YouTube provide inadequate information regarding ERCP. Considering the unique characteristics of this procedure, professionals and academic societies need to be vigilant and proactive in producing and promoting high-quality videos.

## 1. Introduction

With the development and widespread use of the Internet, accessing health information online has become a common practice nowadays.^[1]^ Recent surveys have demonstrated that 80% of Internet users access medical information online.^[2]^ YouTube is one of the most popular video platforms in the world, with over one billion users watching more than one billion hours of content daily.^[3]^ However, due to minimal content guidelines and the lack of a peer-review system, anecdotal or personal opinions can be posted to YouTube. Inappropriate medical information can adversely affect patients’ wellness or their prognosis. Government agencies and healthcare providers have expressed apprehensions about the quality and veracity of the information available on YouTube.^[4,5]^ Moreover, medical experts have acknowledged its significance to the general public as a resource for medical information, and there has been an increase in the number of studies validating the quality of the information in medical videos.^[6]^

Endoscopic retrograde cholangiopancreatography (ERCP) is an advanced endoscopic procedure using endoscopy and fluoroscopy for diagnosing and treating pancreaticobiliary diseases. With advancements in imaging modality, such as magnetic retrograde cholangiopancreatography, ERCP has changed from a diagnostic modality to a therapeutic one.^[7]^ Therefore, it is different from an endoscopy or colonoscopy, which are conducted for the healthy population as part of cancer screening.^[8]^ Furthermore, as ERCP carries a higher risk of adverse events than standard endoscopy,^[9]^ it is crucial to provide accurate information about it. However, no studies have yet analyzed the quality of the information on ERCP available on YouTube. This study aimed to identify whether YouTube videos provide appropriate information on ERCP to the general population.

## 2. Materials and Methods

### 2.1. Video selection

The YouTube search was performed on November 30, 2021, using the terms “endoscopic retrograde cholangiopancreatography” and “ERCP,” by two of the authors of this paper (DWK and JSH). Their personal accounts were not used to avoid the influence of their respective video histories. The top 50 results of both searches, sorted by relevance and view count, were collected. They were then filtered according to a set of inclusion and exclusion criteria. The inclusion criteria were that the videos had to be in English and primarily related to ERCP. The exclusion criteria were that they could not be duplicates, case reports for experts (e.g., an educational demonstration of a liver procedure for endoscopists at conferences), conference videos, in languages other than English, lacking audio, and unrelated videos. The institutional board review was not required for this study.

### 2.2. Data extraction

Two reviewers (JSH and HS) extracted data from each video. The authors performing the analysis were gastroenterologists certified by the Korean Association of Internal Medicine. Each video was assessed in terms of the following parameters: the date of upload, running time (seconds), the total number of views, the number of comments, and total numbers of “likes” and “dislikes,” as represented by the “thumbs up” and “thumbs down” icons, respectively. In terms of the type of provider, video sources were categorized as follows: civilian, healthcare professionals (academic centers, hospitals, or physicians), medical websites (foundations or academic journals), and media or TV channels.

### 2.3. Assessment of quality

For quality assessment, two authors (SWK and HS) created a scoring system called “ERCP Data Quality score (E-DQS),” a modified form of Colonoscopy Data Quality score (C-DQS), a scoring system designed to evaluate the quality of colonoscopy videos.^[10]^ E-DQS consists of 17 statements comprising the definition of ERCP and expectations before, during, and after the procedure, and the total score for each video is assigned on a scale from 0 to 20 (Table 1). We also evaluated the global quality score (GQS, a validated score system for rating the overall quality of healthcare videos)^[11]^ of each video (Table 2). In case of discrepancies between the scores assigned by the two authors, the third author (JSH) arbitrated the evaluation.

**Table 1:** ERCP Data Quality Score (E-DQS).

Definitions	Points
Defines RCP (e.g., ERCP as the procedure to diagnose and treat pancreaticobiliary diseases)	1
Defines duodenoscope, a flexible tube with a side-viewing camera and light at the end, which allows observation of ampulla, and access to the biliary or pancreatic duct	1
Indications for ERCP as a diagnostic and therapeutic procedure (1 point for mentioning any of the indications below)	1
Common bile duct stones	
Biliary malignancies	
Ampulla tumors	
Pancreas malignancies	
Sphincter of Oddi dysfunctions	
Expectations before the procedure	
Mentions that the patient will have to give written informed consent form before the procedure	1
Recommends no food or drinks 6 h before the procedure	1
Describes that the doctor will advise ceasing certain medications before the procedure	1
Expectations during the procedure	
Mentions that the procedure will be performed under sedation	1
Describes that patients will be irradiated by fluoroscopy during the procedure	1
Describes that the patients will be treated in the prone position	1
Describes duodenoscope and how it works (e.g., the scope will be passed to the duodenum through the esophagus and stomachMentions selective cannulation to bile or pancreatic ducts through the ampulla with cannula	1
Mentions that contrast agents will be injected through the duodenoscope channel to enhance anatomical image of bile duct or pancreas	1
Describes what endoscopist looks for during the procedure (e.g., common bile duct stones, biliary or pancreatic malignancies, etc.)	1
Describes complications of the procedure (1 point each, maximum 4 points)	4
Pancreatitis	
Perforation	
Bleeding	
Adverse drug reaction	
Describes that certain types of drainage can be performed after the procedure (e.g., ENBD, ERBD, or ERPD)	1
Expectations after the procedure	
Mentions that the patient can feel bloating or abdominal pain after the procedure	1
Describes that follow-up ERCP or another rescue approach (PTBD) can be performed if the index procedure fails	1
Mentions that the duration of fasting after the procedure depends on the type of procedure and the patient’s condition	1

ENBD = endoscopic nasobiliary drainage, ERBD = endoscopic retrograde biliary drainage, ERCP = endoscopic retrograde cholangiopancreatography, ERPD = endoscopic retrograde pancreatic drainage, PTBD = percutaneous transhepatic biliary drainage.

**Table 2:** Global Quality Scale (GQS).

1. Poor quality – poor flow, most information missing, not helpful for patients
2. Generally poor – some information given but of limited use to patients
3. Moderate quality – some important information is adequately discussed
4. Good quality – good flow, most relevant information is covered, useful for patients
5. Excellent quality – excellent flow, useful for patients

### 2.4. Statistical analysis

Baseline characteristics including days since upload, running time, number of views, number of likes and dislikes, and number of comments were noted. Continuous variables were expressed as the mean and range, and categorical variables as frequencies and percentages. Either ANOVA or the Kruskal–Wallis test was used (as indicated) for comparisons between quantitative variables. *P* value < .05 was considered to be statistically significant. All statistical analyses were performed using the R software (R Foundation for Statistical Computing, Vienna, Austria, http://www.R-project.org, Ver 4.1.0).

## 3. Results

### 3.1. Characteristics of videos

**Figure 1.**
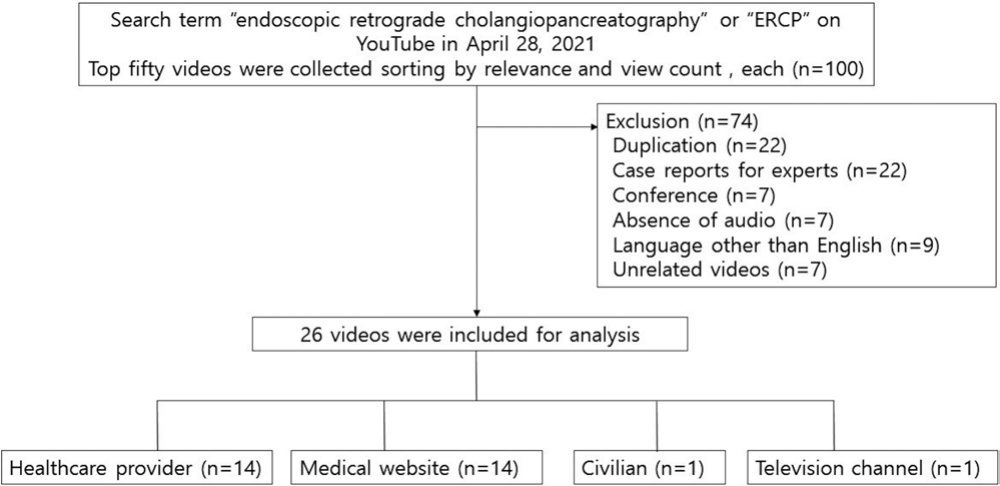
Flow chart of YouTube videos selection for analysis.

Out of the 100 videos collected using the chosen search strings, 74 were excluded in line with the criteria specified above. A total of 26 videos were eligible for the final analysis (Table S1, Supplemental Digital Content, http://links.lww.com/MD/H384). The flow of the study is presented in Figure 1. The 26 videos had a total of 2,135,513 views, with a median of 3318 views per video and a range between 32 and 1,054,067. The videos were uploaded between August 2009 and March 2021. The median period since upload was 2039 days, with a range of 41 to 4276 days. The median running time of the videos was 146 seconds, with a range between 54 and 1323 seconds. The median numbers of likes and dislikes per video were 345 and 2, respectively.

Among all the videos, 14 (53.8%) were uploaded by healthcare professionals (physician or hospital) and 10 (38.6%) by medical websites; only one video was uploaded by a layperson and one by a TV channel. The videos with the most views and maximum likes were featured in an animation describing details of the procedure (youtube.com/watch?v = 5VgoDJ31V_0). The basic characteristics of the included videos are presented in Table 3.

**Table 3:** Basic characteristics of included videos.

No. of videos included in analysis	Time period of uploads	Median days since upload	Median running time of videos	Median no. of views	Median no. of “likes”	Median no. of “dislikes”	Median no. of “comment”
26	08/2009–03/2021	2039 [41–4276]	146 [54–1323]	3318 [32–1,054,067]	345 [0–3200]	2 [0–164]	11 [0–109]

### 3.2. Quality of videos

The overall median E-DQS scores for enrolled videos were low: 6.5 out of 20 (Table 4). The overall median scores against each E-DQS statement type were as follows: definition – 2.4 out of 3 points; expectations before the procedure – 0 out of 3 points; expectations during the procedure – 4 out of 11 points; expectations after the procedure – 0 out of 3 points. The differences between scores by video provider were not statistically significant (*P* = .420). The distribution of GQS for all videos analyzed was as follows: poor quality – 5 (19.2%); generally poor – 10 (38.5%); moderate quality – 6 (23.1%); good quality – 2 (7.7%); excellent quality – 3 (11.5%). GQS according to authorship was also not statistically significant (*P* = .170).

**Table 4:** Video quality distribution according to upload source.

	Upload source				Total	*P*
	Civilian	Medical website	Physician or Hospital	TV channel		
Videos, n (%)	1 (3.8)	10 (38.6)	14 (53.8)	1 (3.8)		
E-DQS						
Definition	3.0	2.0 [2.0; 2.0]	3.0 [2.0; 3.0]	1.0	2.4 [2.0–3.0]	.287
Before	3.0	0.5 [0.0; 1.0]	0.0 [0.0; 1.0]	0.0	0.0 [0.0–1.0]	.204
During	10.0	3.5 [2.0; 5.75]	3.0 [1.0; 3.0]	5.0	4.0 [2.0–5.0]	.100
After	0.0	0.0 [0.0; 0.0]	0.0 [0.0; 1.0]	0.0	0.0 [0.0–0.0]	.688
Total	16.0	6.0 [3.0; 10.0]	6.0 [4.0; 8.0]	6.0	6.5 [4.0–9.5]	.420
GQS, n (%)						
Poor quality	0 (0.0)	2 (20.0)	3 (21.4)	0 (0.0)	5 (19.2)	.170
Generally poor	0 (0.0)	3 (30.0)	6 (42.9)	1 (100.0)	10 (38.5)	
Moderate quality	0 (0.0)	4 (40.0)	2 (14.3)	0 (0.0)	6 (23.1)	
Good quality	1 (100.0)	0 (0.0)	1 (7.1)	0 (0.0)	2 (7.7)	
Excellent quality	0 (0.0)	1 (10.0)	2 (14.3)	0 (0.0)	3 (11.5)	

Values are reported as median [interquartile range] unless indicated otherwise.

E-DQS = ERCP data quality score, GQS = global quality scale.

## 4. Discussion

This study analyzed 26 YouTube videos on ERCP. The quality was evaluated using GQS and the E-DQS created by the authors. Low E-DQS and GQS scores demonstrated that ERCP videos on YouTube were insufficient as sources of information. Although most of the analyzed videos are produced by medical websites, physicians, or hospitals, the overall quality was quite low, with the median of total E-DQS being 6.5 out of 20 (Table 4). Specifically, expectations before and after the procedure were rarely described in the analyzed videos. However, low scores in these two areas may not be significant in terms of evaluating the quality of the ERCP videos, since the statements assessing expectations are almost the same as those used in the case of colonoscopy (C-DQS).^[10]^ Therefore, it is pivotal to investigate the scores for expectations during the procedure, which touch on essential aspects of the ERCP.

The median score for expectations during the procedure for all videos was 4 out of 11, and the scores for videos posted by medical websites and physicians/hospitals were 3.5 and 3, respectively. The low score was due to the large number of videos that did not describe the unique features of ERCP; only 13 out of 26 videos informed viewers that patients would be irradiated by fluoroscopy and that contrast agents would be used. Moreover, only six videos described at least one adverse event related to ERCP.

The highest ranked video source (E-DQS – 16 points) was posted by a layperson (https://www.youtube.com/watch?v=6ukxYaw9NE8). He explained the various aspects of ERCP as well as possible complications using a slide presentation format, radiographs, and an anatomical atlas. However, this video only had 77 views and no likes; the lack of attention might be attributed to the frequent use of medical jargon and excessive playback time (22 minutes). The use of difficult vocabulary and radiographs might make it challenging for people to understand the information about ERCP, in contrast with videos using intuitive animations. The video with the highest number of likes was uploaded by a medical website (https://www.youtube.com/watch?v=5VgoDJ31V_0), and has the second highest number of views. The video comprised animation, text, and a voiceover. The definition of ERCP, the indications for the procedure, the preparation process before the procedure, the procedure, and the related complications were explained in clear video quality and in an appropriate running time of 7 minutes and 35 seconds. Healthy people have little chance of experiencing ERCP, unlike a colonoscopy. Furthermore, the former is associated with the highest rate of adverse events among endoscopies,^[12]^ with a morbidity rate of 0.08% to 9.7% and a mortality rate of 0.04% to 0.7%.^[13]^ Therefore, we concluded that healthcare professionals and academic societies dealing with ERCP should make an effort to create videos with high-quality animations, simple language and acceptable running time to explain various aspects of and provide accurate information on ERCP.

Internet media platforms such as YouTube are like two-edged swords. On the one hand, people can access medical information from their desks or cell phones without visiting a hospital or physicians. However, misinformation in invalidated videos created by non-experts can affect people’s health drastically. None of the included videos provide incorrect information about the procedure, but most of them missed essential information. Consistent with current trends on social media, medical professionals should pay attention to media sources on the Internet and efforts should be made to cooperate with professionals in the media to produce and validate high-quality videos.

This study has several limitations. First, evaluating the quality of videos could be regarded as a subjective exercise; however, we tried to assign scores objectively, using a preexisting scoring system. Second, the search results are limited by the search date; YouTube is a dynamic website and the videos are likely to change over time. Finally, our study only included videos in English. Hence, the result cannot be applied to videos in other languages.

In conclusion, it may be said that ERCP videos on YouTube provide inadequate information regarding ERCP. Considering the unique characteristics of this procedure, professionals and academic societies need to be vigilant and proactive in producing and promoting high-quality videos about it.

## Author contributions

**Study concept and design:** S.W.K. and H.S.

**Data acquisition:** D.W.K. and J.S.H.

**Data analysis and interpretation:** D.W.K. and J.S.H.

**Drafting of the manuscript:** D.W.K. and S.W.K.

**Critical revision of the manuscript for important intellectual content:** S.W.K. and H.S.

**Statistical analysis:** S.W.K.

All authors have read and agreed to the published version of the manuscript.

**Conceptualization:** Hoonsub So, Sung Woo Ko.

**Data curation:** Do Won Kim, Jun Seong Hwang.

**Formal analysis:** Do Won Kim.

**Supervision:** Hoonsub So.

**Validation:** Hoonsub So, Jun Seong Hwang.

**Visualization:** Jun Seong Hwang.

**Writing – original draft:** Do Won Kim, Sung Woo Ko.

**Writing – review & editing:** Sung Woo Ko.

## Supplementary Material


