# Supplementary material for: YouTube as a source of information on endoscopic retrograde cholangiopancreatography
Source: Medicine (Baltimore). 2022 Sep 23;101(38):e30724. doi: 10.1097/MD.0000000000030724 (PMC9509111; doi:10.1097/MD.0000000000030724)
Supplement: Supplementary file 1 [file medi-101-e30724-s001.pdf]

Supplementary table 1. Included videos for quality analysis

Number	Title	URL
1	Understanding ERCP (Endoscopic Retrograde Cholangiopancreatography)	https://www.youtube.com/watch?v=5VgoDJ31V_0
2	Endoscopic retrograde cholangiopancreatography (ERCP)	https://www.youtube.com/watch?v=IRdA2krJ6LQ
3	Endoscopic Retrograde Cholangiopancreatography (ERCP)	https://www.youtube.com/watch?v=182NjHKjUao
4	The Role of Endoscopic Retrograde Cholangiopancreatography (ERCP)	https://www.youtube.com/watch?v=HnhhRM8r770
5	Pediatric ERCP | Cincinnati Children's	https://www.youtube.com/watch?v=5I504enWYCY
6	Endoscopy Procedures | Endoscopic Retrograde Cholangiopancreatography (ERCP)	https://www.youtube.com/watch?v=GuePY-3ccOg
7	Endoscopic Retrograde Cholangiopancreatography (ERCP) | El Camino Health	https://www.youtube.com/watch?v=BTRFoKgUPrE
8	Endoscopic Retrograde Cholangiopancreatography (ERCP)	https://www.youtube.com/watch?v=Uj2i-L5E4Ws
9	What Is Endoscopic Retrograde Cholangiopancreatography (ERCP)? - Matt Johnson, MD	https://www.youtube.com/watch?v=7V2h3q_aKbU
10	ERCP : What to Expect | IU Health	https://www.youtube.com/watch?v=c6bjIpttBps
11	ERCP for Removal of a Stone in the Bile Duct	https://www.youtube.com/watch?v=DSJjcJBmltg
12	ERCP - Endoscopic Retrograde Cholangiopancreatography	https://www.youtube.com/watch?v=6ukxYaw9NE8
13	Endoscopic Retrograde Cholangiopancreatography (ERCP) & Endoscopic Ultrasound (EUS) at IGH	https://www.youtube.com/watch?v=O7adhUVAsl0
14	What is ERCP?	https://www.youtube.com/watch?v=XxElxaYhElU
15	Endoscopic Retrograde Cholangiopancreatography | Manipal Hospitals India	https://www.youtube.com/watch?v=8V9pV_SVW2k
16	Endoscopic Retrograde Cholangiopancreatography	https://www.youtube.com/watch?v=BkqPLSGYf-A
17	ERCP with sphincterotomy	https://www.youtube.com/watch?v=VeuKnsyymb8
18	Understanding ERCP and scope safety	https://www.youtube.com/watch?v=GnaaL_blhME
19	ERCP	https://www.youtube.com/watch?v=WCgj5ntlvZ0
20	What are the risks of ERCP?	https://www.youtube.com/watch?v=FvXmzD_SMhs
21	Gastro Talk: What is ERCP?	https://www.youtube.com/watch?v=GJrJCCISzj8

22	ERCP endoscopy of bile & pancreatic ducts	https://www.youtube.com/watch?v=izT327nGR8g
23	How is ERCP performed?	https://www.youtube.com/watch?v=slc_K8cu68I
24	ERCP Procedure	https://www.youtube.com/watch?v=vnAtuG9ULB4
25	ERCP with stenting	https://www.youtube.com/watch?v=mp1Tj3VWAJs
26	What follow up care is required after an ERCP?	https://www.youtube.com/watch?v=l09ZCvbFjvw

ERCP = endoscopic retrograde cholangiopancreatography, URL = uniform resource locator
